# A Comparative Study of Otolith Microchemical Characteristics of *Coilia nasus* in Poyang Lake Under Extreme Climate and Normal Periods

**DOI:** 10.3390/ani16172693

**Published:** 2026-08-29

**Authors:** Taoyuan He, Shubin Lin, Tao Jiang, Ruipeng Zhang, Sheng Wang, Legen Peng

**Affiliations:** 1 Wuxi Fisheries College, Nanjing Agricultural University, Wuxi 214081, China; 2022813036@stu.njau.edu.cn (T.H.); lin02292253@163.com (S.L.); 2 Freshwater Fisheries Research Center, Chinese Academy of Fishery Sciences, Wuxi 214081, China; 3 School of Marine Science and Environment Engineering, Dalian Ocean University, Dalian 116023, China; zhang1387915@163.com; 4 Aquatic Conservation and Rescue Center of Jiangxi Province, Nanchang 330096, China; wangsheng_86@163.com (S.W.); peng_legen@163.com (L.P.)

**Keywords:** *Coilia nasus*, otolith microchemistry, Poyang Lake, extreme climate

## Abstract

Poyang Lake, China’s largest freshwater lake, is a critical spawning ground for the anadromous fish *Coilia nasus*. In 2022, extreme drought and upstream regulation caused historically low water levels, exposing lakebeds and desiccating spawning grounds, with spawners found dead. To assess impacts on migration, we collected 11 desiccated and decomposed specimens from the 2022 exposed lakebed and 18 fresh specimens from the same area in 2024 (normal year). Otolith Sr/Ca ratios reliably recorded lifetime salinity. Despite degradation, the 2022 otoliths preserved complete habitat records, confirming method reliability. Most specimens were the migratory long-jaw form of the species; two were resident short-jaw specimens, with uniformly low Sr/Ca ratios. All migrants showed a three-phase anadromous pattern: freshwater hatching (Sr/Ca < 3), marine feeding (>3), and spawning migration into freshwater (<3). The outermost freshwater zone (SIII) was significantly wider in the 2022 migrants (144 ± 30 μm) than in the specimens from 2024 (33 ± 24 μm, *p* < 0.01), indicating that the extremely low water levels severely slowed upstream migration. Critically, driven by natal homing instincts, the fish reached the desiccated sites and did not seek alternative locations, causing mass mortality—revealing high site fidelity and low adaptability. For the planned Poyang Lake Water Conservancy Hub, we recommend maintaining minimum ecological water levels to prevent spawning ground desiccation and ensuring river–lake connectivity during migration and larval dispersal to sustain *C. nasus* and other aquatic animals under warming and engineering pressures.

## Simple Summary

Poyang Lake is a key spawning ground for anadromous *Coilia nasus*. In 2022, extreme drought caused historically low water levels, exposing lakebeds and killing many spawners. We collected 11 desiccated and even decayed specimens from the dry lakebed and compared their otolith microchemistry with that of fresh specimens from 2024 (normal period). Despite decomposition, the otoliths retained complete and accurate habitat records. Both years showed typical freshwater–seawater–freshwater migration patterns, but the 2022 specimens had a significantly wider edge low-salinity zone (SIII: 144 ± 30 μm vs. 33 ± 24 μm), indicating severely slowed upstream migration due to low water levels. Notably, fish did not seek alternative habitats despite reaching desiccated grounds—strong natal homing drove them to the original sites, causing mass mortality. This reveals spawning site fidelity and low adaptability. For the planned Poyang Lake Water Conservancy Hub, we recommend maintaining minimum ecological water levels to prevent spawning ground loss and ensuring river–lake connectivity during migration windows. These measures are critical for population persistence under climate change and engineering pressures.

## 1. Introduction

*Coilia nasus* is widely distributed in the coastal waters and connected rivers of China and has historically been an important target for commercial fisheries [1]. The Yangtze River Basin, as the main distribution area, harbors rich ecological phenotype groups: besides the common anadromous long-jaw anchovy, it also hosts the short-jaw anchovy (*C. brachygnathus*) and the lake anchovy (*C. nasus taihuensis*) [2]. Among these, the anadromous long-jaw anchovy, famously known as the “Yangtze knife fish”, historically migrated as far upstream as Dongting Lake [1], ranking among the “Three Delicacies of the Yangtze” and receiving widespread attention. As a typical anadromous fish, its life history encompasses multiple water types—lake (spawning ground), river (migration corridor/spawning ground), and sea (overwintering/feeding ground)—requiring specific environmental quality in each habitat, while highly depending on the connectivity among them to sustain population recruitment. Thus, the scientific assessment and conservation of *C. nasus* stocks not only concern the survival of this valuable species but also benefit the numerous sympatric aquatic organisms in these habitats. Both in terms of ecological importance and social concern, *C. nasus* is undoubtedly a representative “umbrella species” and a “flagship species” in regards to aquatic wildlife conservation. Therefore, *C. nasus* was selected for this study because it is an iconic anadromous fish that relies on Poyang Lake as a critical spawning ground, is highly sensitive to river–lake connectivity and water-level fluctuations, and can serve as an umbrella species for the conservation of the lake ecosystem.

However, the resource was once perilously depleted due to the superimposed effects of overfishing, critical habitat shrinkage, and blocked habitat connectivity. For example, in the traditional fishing grounds of the Jingjiang section of the Yangtze, the catch per vessel plummeted from 1133.3 kg in 1989 to 66.4 kg in 2009, a decline exceeding 94% [3]. The Poyang Lake waters, historically a traditional spawning ground, were once judged to no longer support anadromous *C. nasus* [4]. Through continuous research, our team confirmed that anadromous *C. nasus* still occurs in Poyang Lake and discovered spawning grounds for the migratory population in the lake area around Lushan City [5]. Subsequent annual surveys monitored the presence of highly fecund spawners in these waters, although the proportion of anadromous *C. nasus* in catches was then only 1.42% ± 2.41% [6]. To fundamentally reverse the “fishless” plight of the Yangtze, a 10-year year-round fishing ban was implemented in 2021, with the issuance of special fishing permits for *C. nasus*, *C. mystus*, and *Eriocheir sinensis* already halted in December 2018. By benefit of these conservation measures, Yangtze *C. nasus* stocks have shown a marked recovery: in the spawning grounds monitored by our team in Poyang Lake, the proportion of anadromous individuals increased to 60.56% ± 40.87% in 2019–2020, a nearly 43-fold rise [6]; the size and resource density of *C. nasus* in the Yangtze Estuary have also rebounded significantly [7,8].

**Figure 1:**
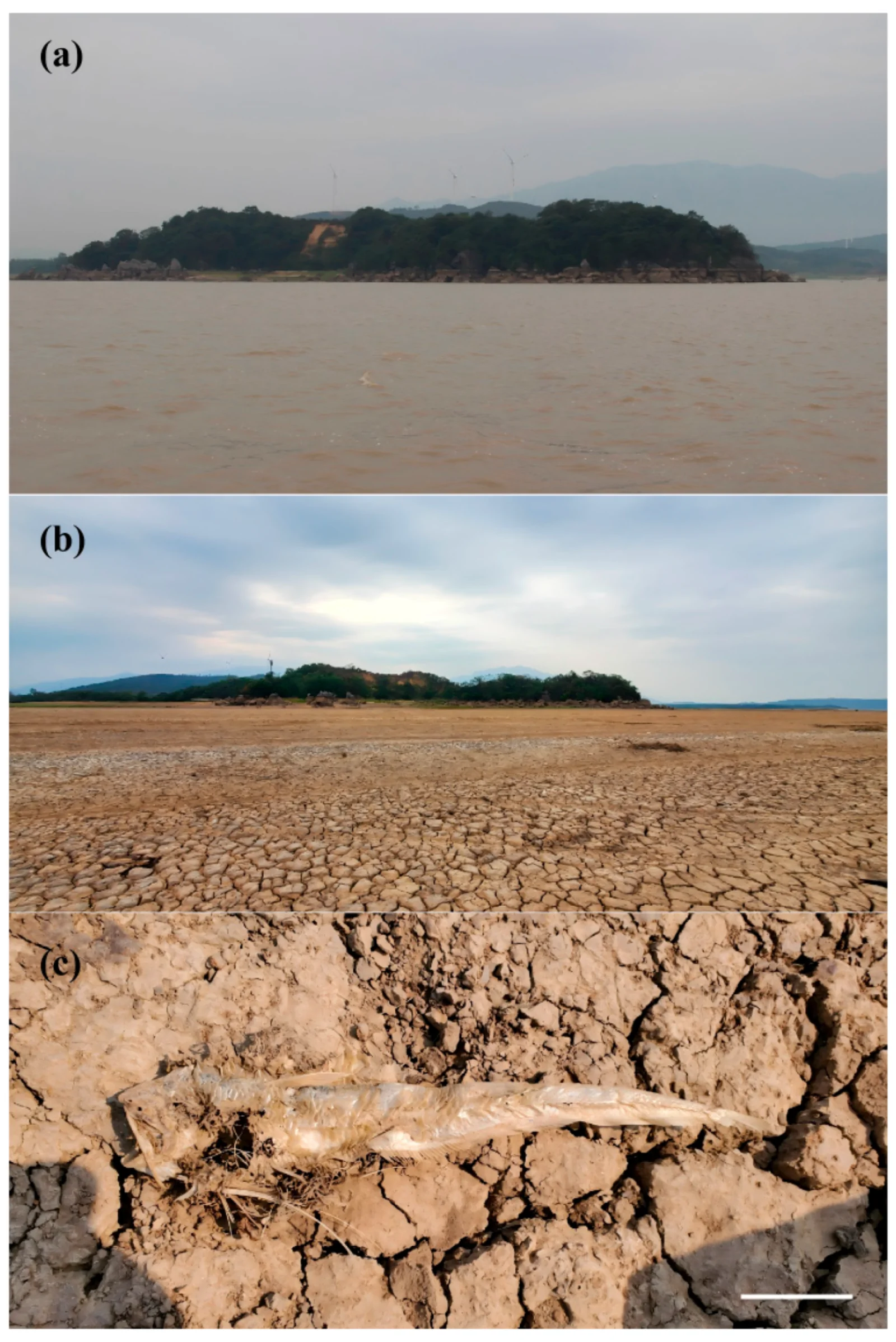
Spawning ground waters and desiccated individuals of *Coilia nasus* in Poyang Lake. (**a**) September 2015, (**b**) September 2022, and (**c**) desiccated individuals in 2022; the scale bar represented 5 cm.

With the deep implementation of the 10-year Yangtze fishing ban, the aquatic biological resources of the Yangtze have shown promising signs of overall recovery [9,10]. Given that fish and other aquatic organisms are highly dependent on critical habitats such as spawning grounds, the next phase of the conservation strategy urgently needs to shift from a fishing ban rescue approach to precise habitat restoration [11]. In this context, Poyang Lake, as the core spawning ground for the *C. nasus* migratory population, is particularly critical in terms of habitat quality and stability. Notably, even with a clear resource recovery and the reappearance of migratory individuals in historical distribution areas, the Poyang Lake waters remained a key habitat for the Yangtze migratory *C. nasus* population—especially the waters where spawning grounds were concentrated [12,13]. Meanwhile, an increasing number of studies have revealed that as a long-distance migratory fish, *C. nasus* exhibits significant “natal homing” behavior [13,14], making Poyang Lake irreplaceable for the reproduction of the migratory population. However, since the impoundment of the Three Gorges Reservoir, Poyang Lake’s water level has been persistently low. The 2022 drought was the most intense, longest-lasting, and most extensive since systematic observations began in 1956 [15]. This event caused extensive lakebed exposure and the desiccation of some anadromous *C. nasus* spawning grounds (Figure 1). During surveys, multiple desiccated *C. nasus* carcasses were found on the dry lakebed. It was evident that extreme climate also significantly impacted the upstream migration of spawners. Given the advantages of otoliths in terms of mineralization stability [16] and their ability to objectively record fish migratory histories [17,18,19,20,21,22,23], this study aimed to use otolith microchemistry to reveal the impact of extreme climate on the migration of *C. nasus* in Poyang Lake.

Due to their high physiological and chemical stability, otoliths can objectively record the migratory history and habitat characteristics of an individual fish during its lifetime, and they have even been applied in archaeological research [24,25,26]. They have been proven to be one of the most effective tools for studying the migratory ecology of *C. nasus* [14,27,28,29,30]. Based on this, the specific objectives of this study were to: (1) validate the reliability of otolith Sr/Ca microchemistry in desiccated and decomposed specimens by comparing it with the results for fresh specimens; (2) reconstruct and compare the migratory histories of anadromous *C. nasus* between the extreme drought year (2022) and a normal hydrological year (2024); and (3) evaluate whether natal homing behavior constrained individuals to desiccated spawning grounds and discuss the implications for the planned Poyang Lake Water Conservancy Hub.

It is particularly emphasized that although this study was based on dried specimens collected during the 2022 extreme low-water event, otolith microchemistry constitutes a permanent elemental fingerprint record; neither dried preservation nor the passage of time would alter its information. These specimens authentically and accurately “freeze-framed” the immediate ecological response to a historically extreme hydrological event. Against the backdrop of superimposed global warming–drying and watershed engineering regulation effects, such non-reproducible natural archives provided extremely scarce and irreplaceable empirical data for understanding the behavioral responses of *C. nasus* to the loss of critical habitat function and for predicting population responses and persistence under future aridification scenarios. Furthermore, this study also provided critical information for the planning and future ecological regulation of the proposed Poyang Lake Water Conservancy Project.

## 2. Materials and Methods

### 2.1. Fish Samples

**Figure 2:**
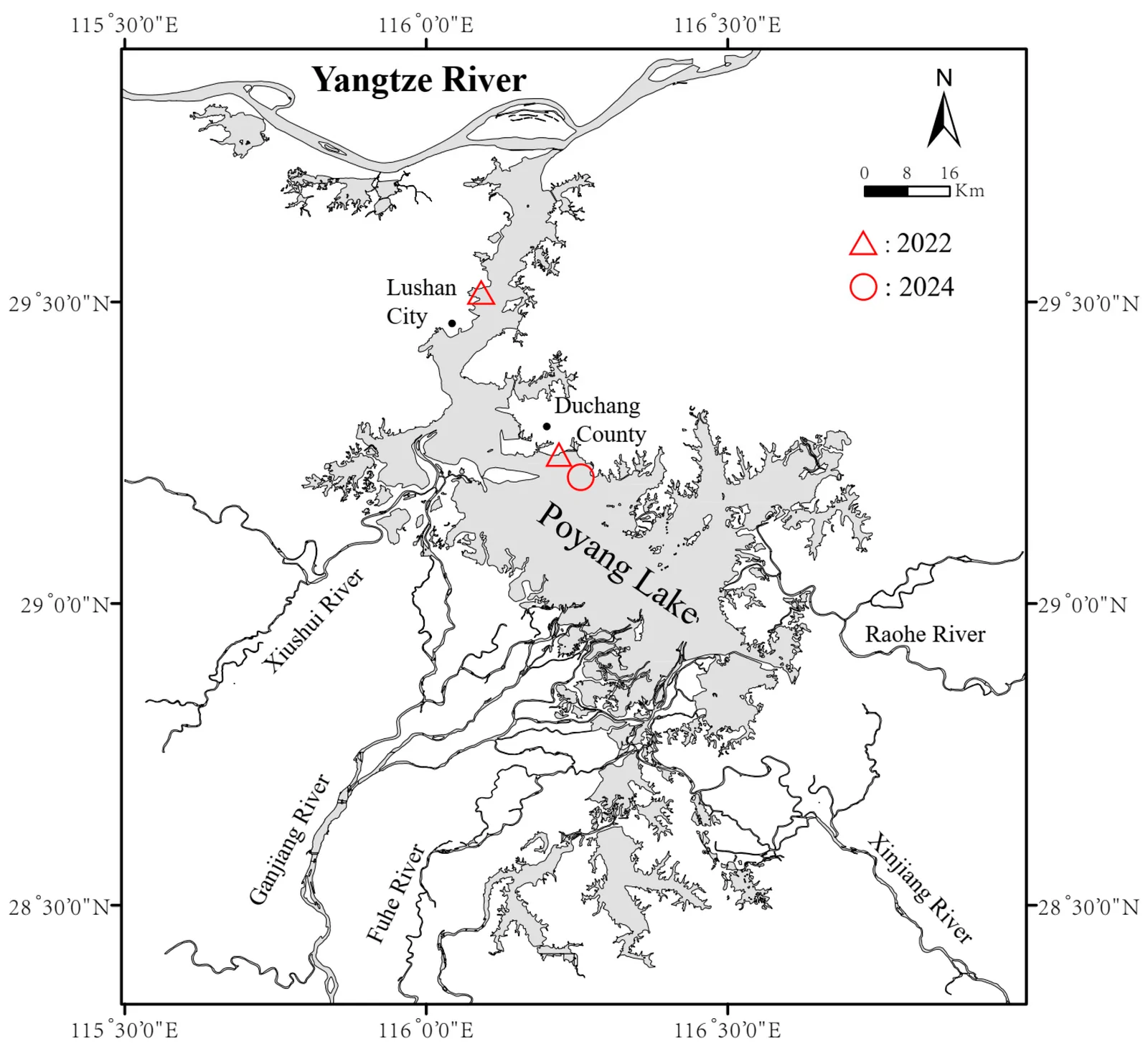
Sampling site of *Coilia nasus*.

The desiccated and even decayed *C. nasus* specimens were collected from the lakebed around Lushan City (2 individuals) and the southern lakebed of Duchang County (9 individuals) in 2022 (Figure 1c and Figure 2). Additionally, fresh specimens (18 individuals) were collected from the southern waters of Duchang County in 2024, with permission from the local Department of Agriculture and Rural Affairs (Figure 2). During the sampling period, this site was situated within the lacustrine zone, with a water depth of approximately 5 m. Based on maxilla characteristics, among the dried specimens, one short-jaw anchovy (maxilla length/head length < 1) was found from each of the Lushan and Duchang waters; all other dried and fresh specimens were typical long-jaw anchovy (maxilla length/head length > 1, Table 1).

### 2.2. Otolith Microchemical Analysis

After the specimens were transported to the laboratory and basic biological data were measured, sagittal otoliths were extracted for microchemical analysis. The otolith preparation and analysis procedures followed those of previous studies [14]. The largest sagittal plane was selected as the analysis surface. Prior to microchemical analysis, the sample surfaces were coated with carbon film using a vacuum carbon evaporator (JEE-420, JEOL Ltd.; Tokyo, Japan) at 36 A for 25 s to enhance conductivity. An electron probe microanalyzer (EPMA, JXA-8100, JEOL Ltd.; Tokyo, Japan) was used to analyze the Sr and Ca elemental concentration profiles along the longest radius from the core to the edge, as well as the Sr distribution on the sagittal plane of representative individuals. The analytical conditions were as follows: accelerating voltage, 15 kV; electron beam current, 20 nA (quantitative analysis) and 500 nA (mapping analysis); dwell time, 15 s (quantitative analysis) and 30 ms (mapping analysis); beam spot diameter, 5 μm; and quantitative point analysis intervals of 20 μm from the core to the edge. Strontium titanate (SrTiO_3_) and calcium carbonate (CaCO_3_) were used as standards. After analysis, the otolith samples were re-polished to remove the carbon film and then etched with 5% EDTA to reveal annual rings for age determination. To assess possible diagenetic alteration, otoliths from both 2022 and 2024 specimens were repolished and examined under a scanning electron microscope (SEM, Pure-SED, Thermo Fisher Scientific, Eindhoven, Netherlands) to check for any signs of dissolution or recrystallization.

### 2.3. Statistical Methods

Conventionally, the quantitative results were ultimately processed as strontium-to-calcium ratios (Sr/Ca × 1000). According to previous studies, the Sr/Ca ratio ranges for *C. nasus* otoliths corresponding to freshwater, estuarine brackish water, and seawater habitats are <3, 3–7, and >7, respectively [5], with the corresponding Sr mapping results appearing blue, green-to-yellow, and red [14,27]. To discuss population characteristics, the freshwater coefficient (*Fc*) was calculated following the methods of a previous study [14], using the formula:

*Fc* = *L_f_*/*L_T_*, (1)

where *Fc* is the freshwater coefficient, *L_f_* is the length of a continuous low Sr/Ca zone (<3) starting from the otolith core, and *L_T_* is the total length from the core to the edge measured during analysis. Statistical calculations and graphical plots were performed using R (4.4.2). Comparisons of differences were conducted using the Mann–Whitney *U*-test. The Mann–Whitney *U*-test is nonparametric and remains valid for unequal sample sizes; however, because the drought-year group was limited to 11 individuals, the statistical power was reduced, and the resulting comparisons should be interpreted as indicative rather than definitive. This limitation is further addressed in the Discussion section.

## 3. Results

The *C. nasus* specimens in this study were predominantly 3 years old, on average, with the 2022 specimens aged 2 to 3 years, making them slightly younger than the 2024 specimens (aged 3–4 years). Comparing the characteristics of long-jaw anchovy individuals between the two years, the total length of the 2022 individuals (311 ± 21 mm) was shorter than that of the 2024 individuals (333 ± 19 mm) (*p* < 0.05).

**Figure 3:**
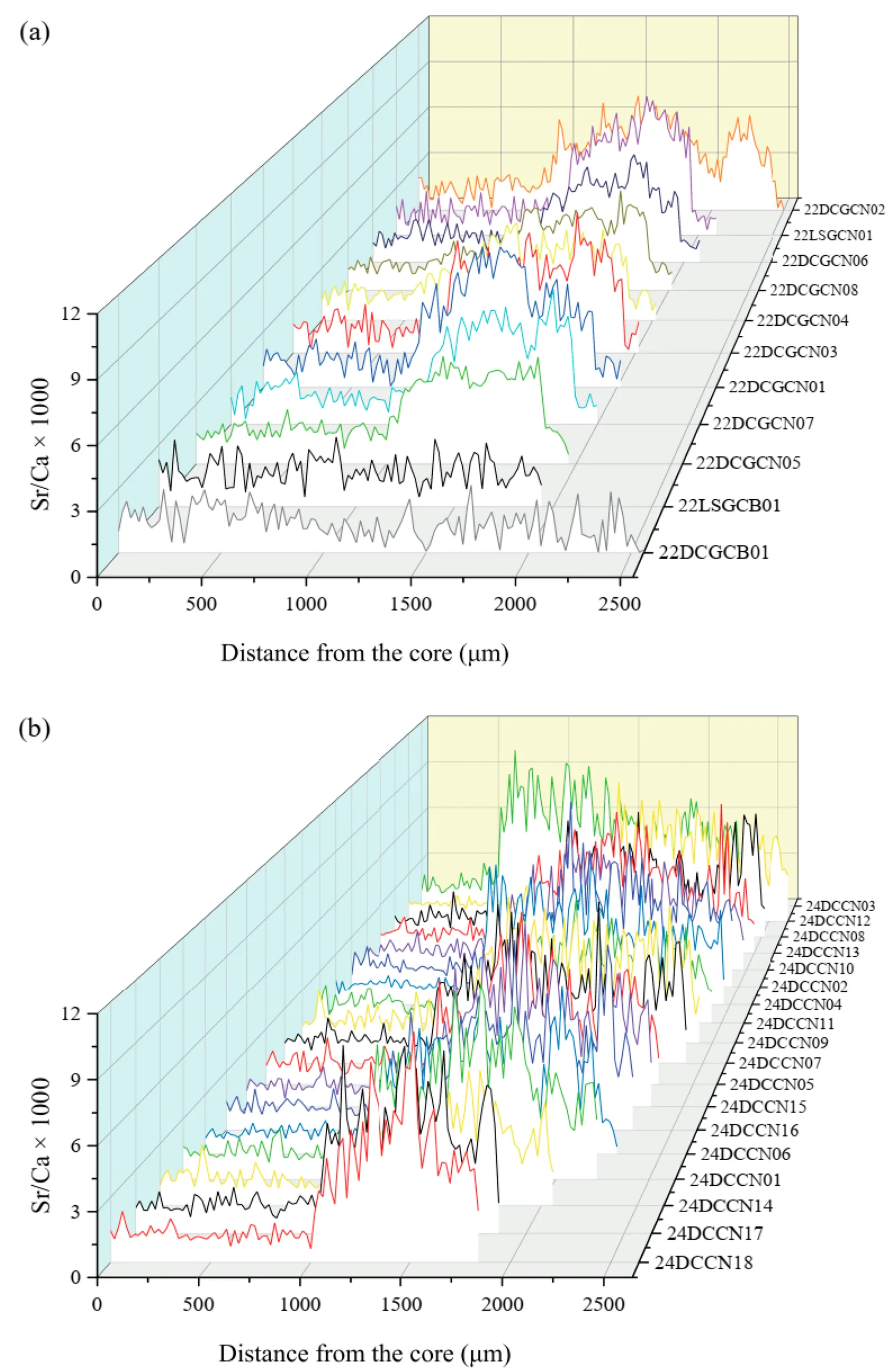
Variations in otolith Sr/Ca ratios (Sr/Ca × 1000) along the longest radius from the core to the edge for anadromous *Coilia nasus* collected in (**a**) the extreme drought year 2022 (dried specimens, *n* = 11) and (**b**) the normal year 2024 (fresh specimens, *n* = 18). Lines of distinct colors represent distinct individuals.

**Figure 4:**
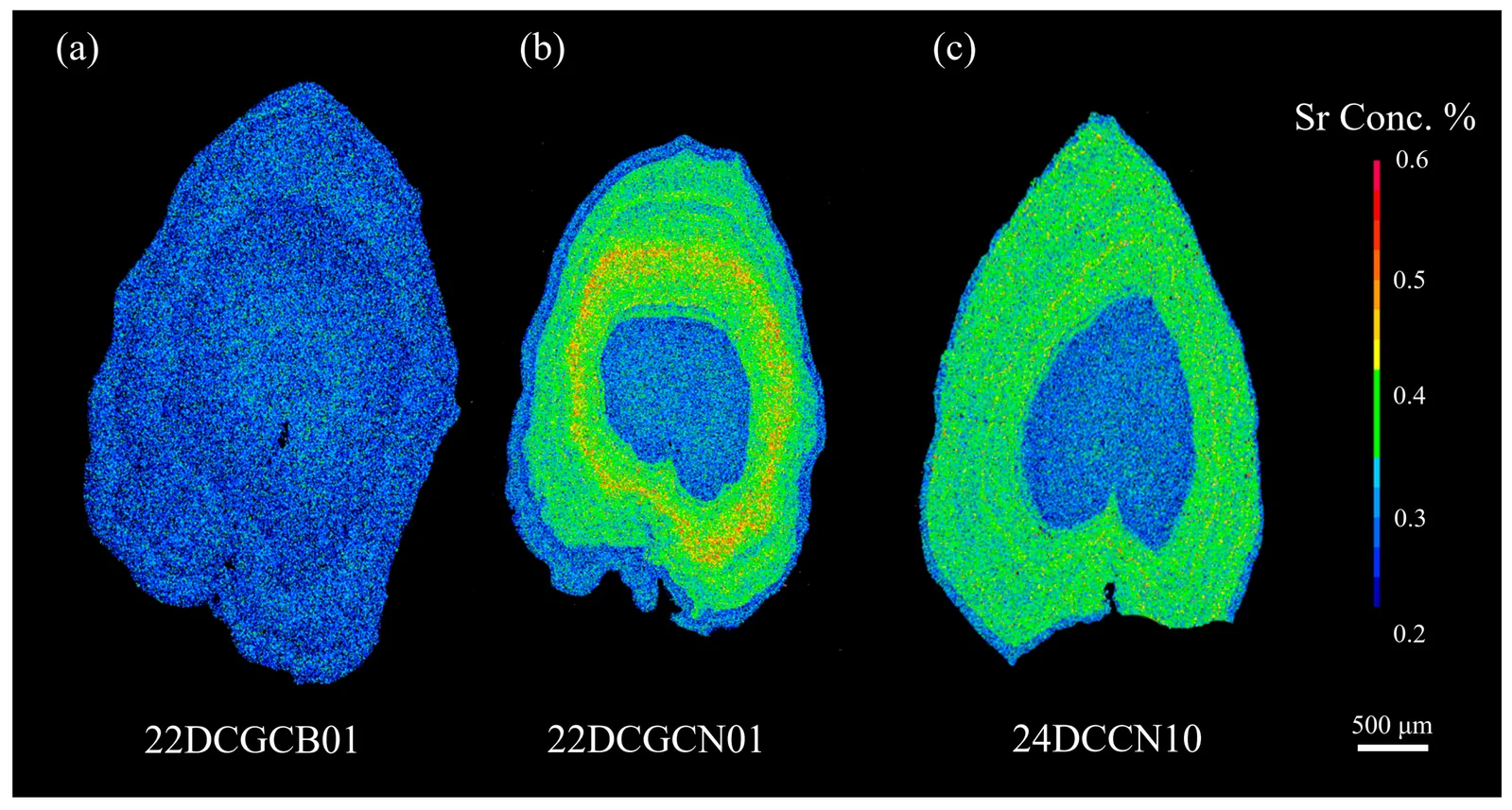
Sr content maps (EPMA) of representative sagittal otoliths from *Coilia nasus*: (**a**) short-jaw resident individual 22DCGCB01; (**b**) anadromous long-jaw individual 22DCGCN05; (**c**) anadromous long-jaw individual 24DCCN01. Color scale from blue (low Sr/Ca, freshwater) through green/yellow (brackish) to red (high Sr/Ca, seawater).

**Figure 5:**
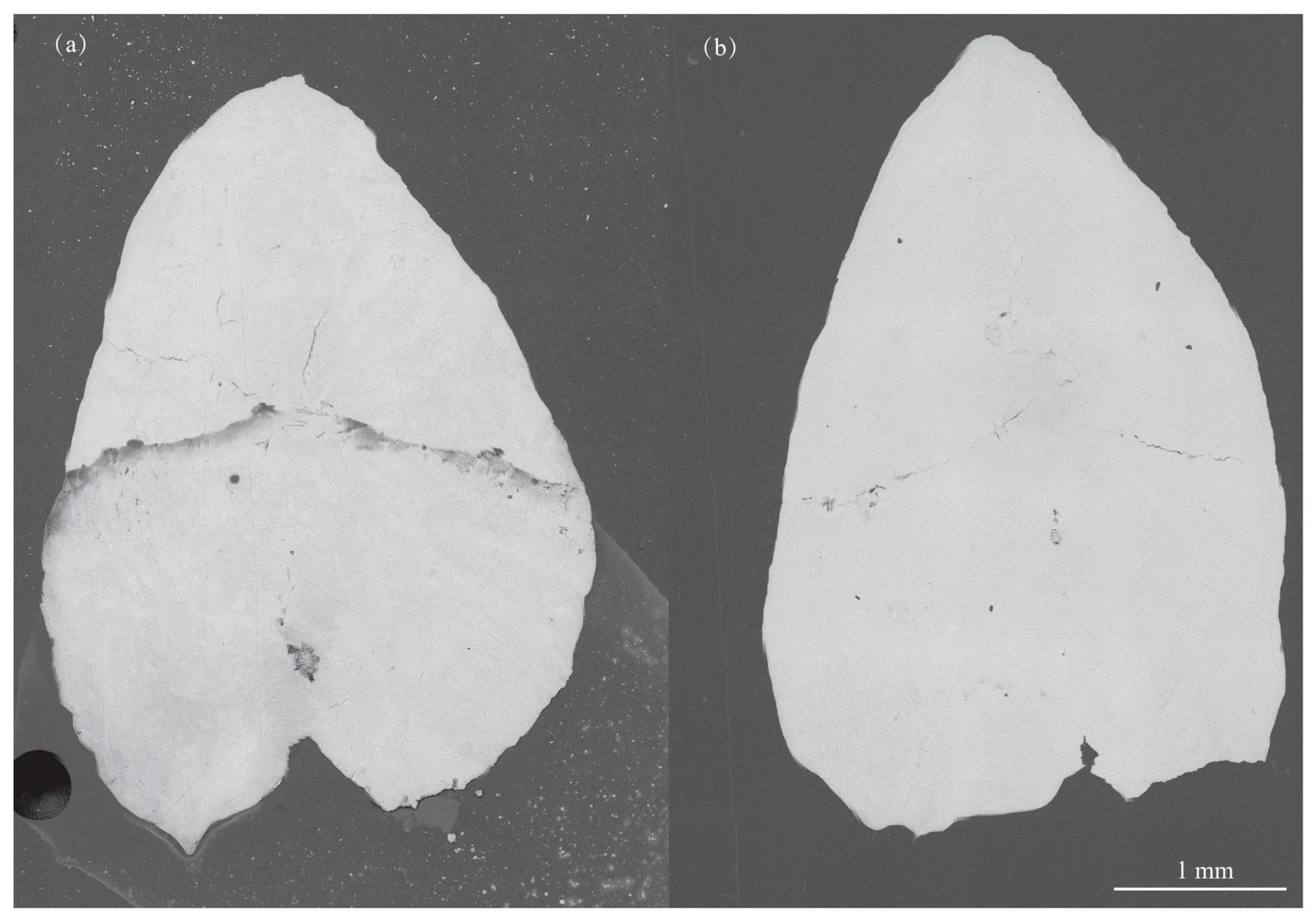
Scanning electron microscope (SEM) images of otoliths from drought fish collected in 2022 ((**a**) 22DCGCN02) and fresh fish collected in 2024 ((**b**) 24DCGCN05) of *Coilia nasus*, showing intact growth increments and no obvious dissolution or recrystallization.

Otolith Sr/Ca ratio results showed that the two short-jaw anchovies, 22DCGCB01 and 22LSGCB01, exhibited ratios of 1.37 ± 0.75 and 1.73 ± 0.69, respectively, consistently below 3. The remaining long-jaw anchovy individuals (22DCGCN01–08, 22LSCN01, and 24DCCN01–18) each displayed three distinct phases from the core to the edge. For the 2022 individuals, these were exhibited a low-value zone SI from 0 to 840–1100 μm (1.35 ± 0.63~1.80 ± 0.59), a middle high-value zone SII (1840–2420 μm) of 4.09 ± 0.80~6.44 ± 1.09, and an edge low-value zone SIII (1960–2500 μm) of 0.90 ± 0.60~1.40 ± 0.49 (*p* < 0.01, Figure 3a). For the 2024 individuals, the corresponding three phases included a core low-value zone SI (0 to 560–1260 μm, 1.06 ± 0.28~1.51 ± 0.50), a middle high-value zone SII (1820–2620 μm, 3.76 ± 1.16~5.71 ± 1.75), and an edge low-value zone SIII (1840–2640 μm, 1.31 ± 0.57~2.95), with significant differences among the phases (*p* < 0.01, Figure 3b). In all three zones, SI and SIII were <3, and SII was >3. The SI phase length (998 ± 165 μm) was significantly longer than the SIII phase (70 ± 59 μm, *p* < 0.01, Figure 3). The Sr concentration maps of typical individuals showed that the sagittal planes of short-jaw anchovies were uniformly blue with low values, while the migratory individuals displayed a clear pattern from core to edge: a blue low-value core zone (SI), a green-to-red high-value zone (SII), and a blue low-value edge zone (SIII, Figure 4). Furthermore, surface examination of otoliths from 2022 and 2024 specimens under an SEM showed intact growth increments and no obvious dissolution, pitting, or recrystallization (Figure 5).

**Figure 6:**
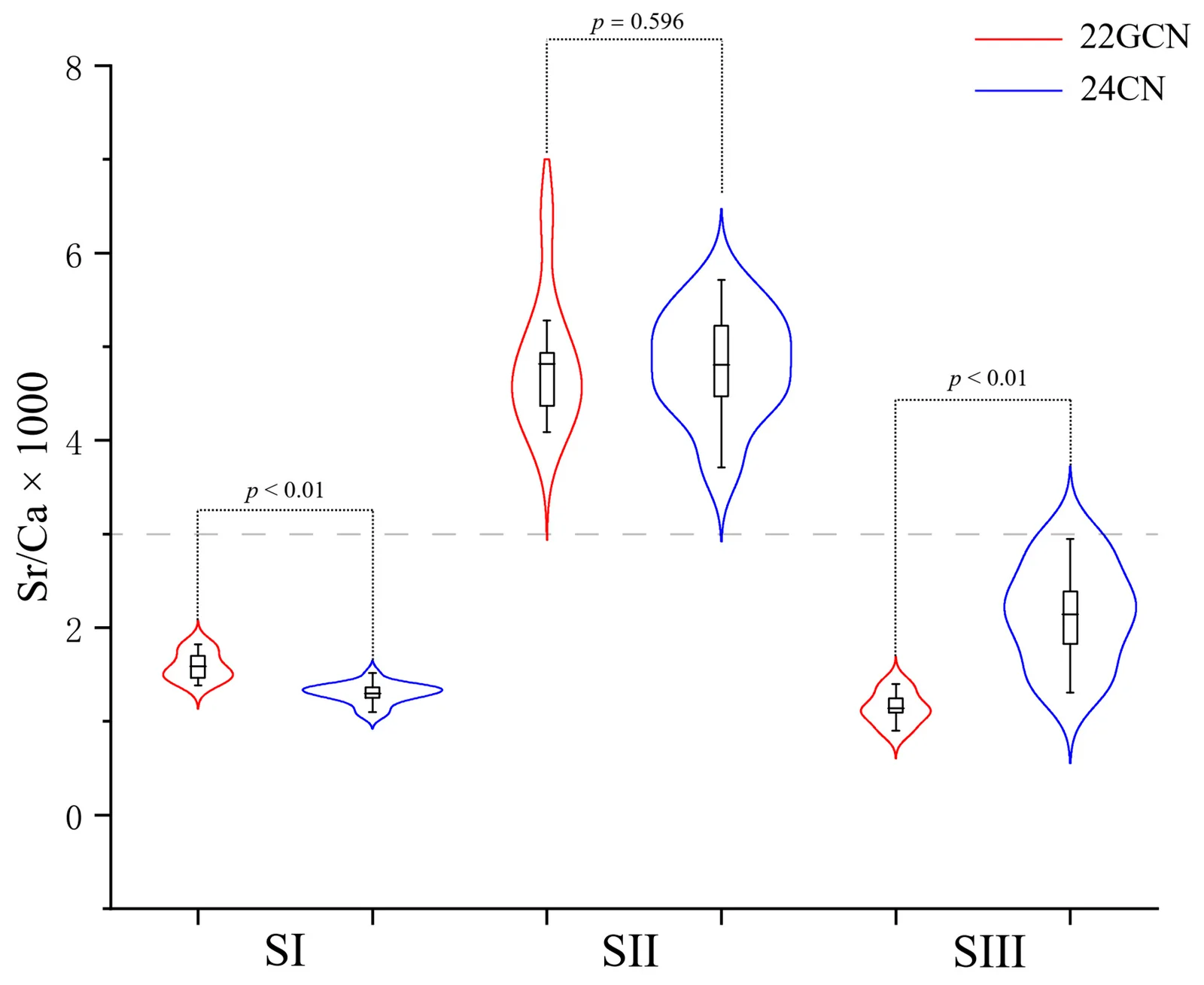
Comparison of otolith Sr/Ca ratios in different phases between 2022 (drought, *n* = 9) and 2024 (normal, *n* = 18) anadromous *Coilia nasus* individuals. SI: core low-value zone; SII: middle high-value zone; SIII: edge low-value zone. Dashed line indicates Sr/Ca × 1000 = 3. In box plots, center lines indicate medians, boxes represent interquartile ranges (IQR), and whiskers extend to 1.5 × IQR. *p* values are from Mann–Whitney *U*-tests.

Comparing the Sr/Ca ratio characteristics across the three phases between the 2022 and 2024 long-jaw anchovies, significant differences existed in SI and SIII between the two-year groups (*p* < 0.01), while the difference in the SII phase was not significant (*p* = 0.596, Figure 6).

**Figure 7:**
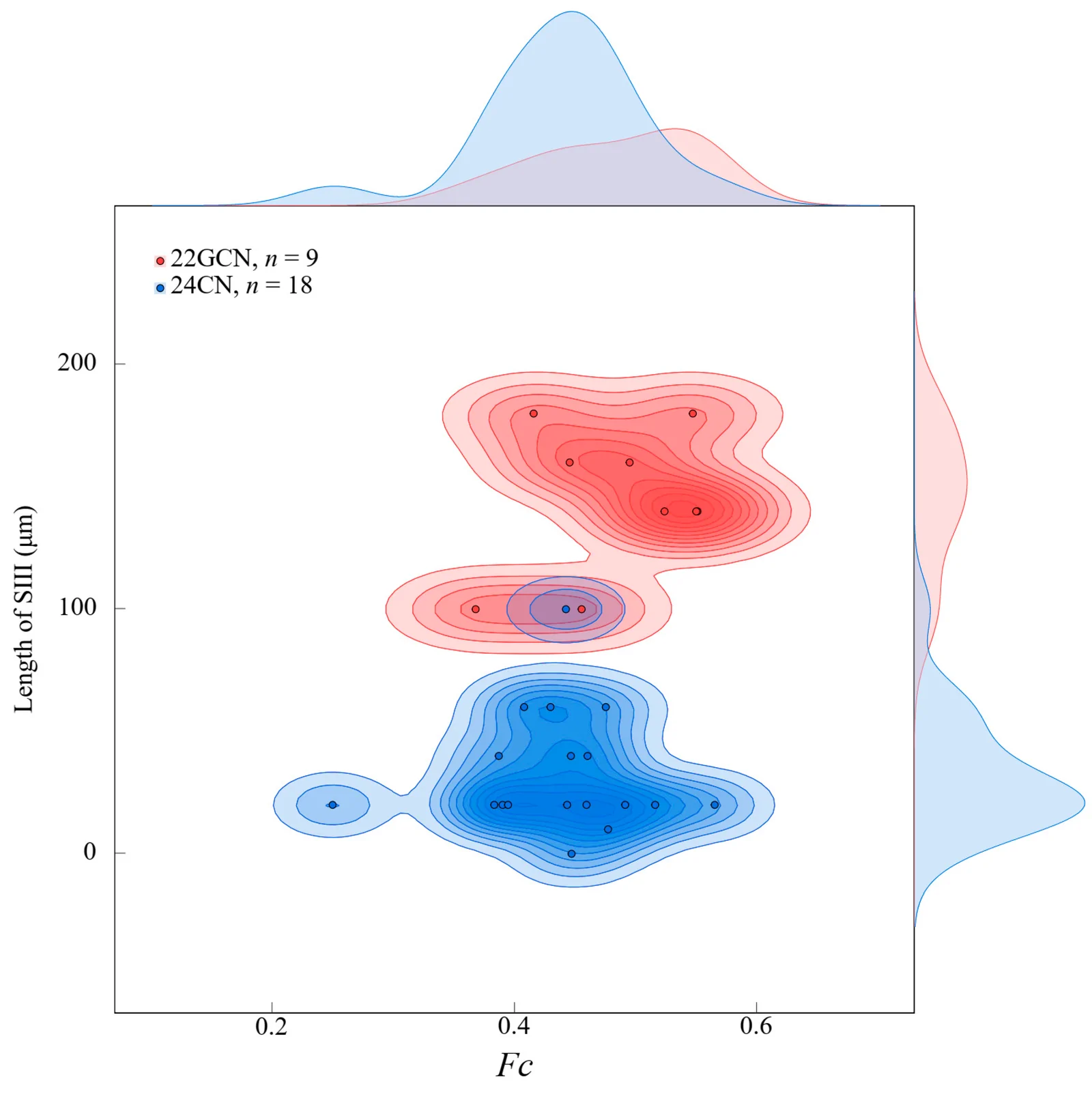
Freshwater coefficient (*Fc*) and SIII extent for anadromous individuals of *Coilia nasus* in 2022 (drought, *n* = 9) and 2024 (normal, *n* = 18).

The freshwater coefficient (*Fc*) results showed that except for the short-jaw anchovies, which had an *Fc* of 1, the 2022 long-jaw anchovies had *Fc* values ranging from 0.37 (22DCGCN02) to 0.55 (22DCGCN05), and the 2024 specimens from 0.25 (24DCCN03) to 0.57 (24DCCN18). No significant difference in *Fc* was found between the long-jaw anchovy groups from the two years (*p* = 0.106). It is noteworthy that all 2022 long-jaw anchovy individuals exhibited a very distinct edge low-value zone (SIII) (Figure 3 and Figure 4), with a length of 144 ± 30 μm. In contrast, although one 2024 individual (24DCCN06) had an SIII length of 100 μm, the overall SIII length in the 2024 group (33 ± 24 μm) was significantly shorter than that of the 2022 individuals (*p* < 0.01, Figure 7).

## 4. Discussion

### 4.1. Influence of Desiccated Specimens on Otolith Sr/Ca Ratios

Fish otoliths are primarily composed of aragonite-structured calcium carbonate and possess relatively stable chemical and physiological properties. On one hand, otoliths are metabolically inert within the fish body and are resistant to resorption or decomposition after formation [16]; on the other hand, they are also difficult to degrade post-mortem, hence their frequent use as research materials in archaeology and paleoclimate studies. However, existing research has largely focused on morphological analysis [31,32,33], with insufficient exploration of the applicability of otolith microchemistry to degraded or desiccated specimens. Wang et al. compared ancient and modern otoliths of *Gymnocypris przewalskii* and found significantly elevated Mg/Ca and δ^18^O values in ancient otoliths, reflecting intense evaporation and concentration in past habitats, but that study mainly focused on environmental proxy changes and did not systematically assess the influence of preservation state on trace element records [34]. The incorporation mechanisms of trace elements in otoliths provided a theoretical basis for their stability. Based on element regulation pathways, otolith trace elements could be divided into “tracers of environmental history” (e.g., Sr, Ba) and “tracers of physiology” (e.g., P, Cu, Zn), while Mg and Mn had dual characteristics [35]. In particular, Sr in the sagitta was incorporated into the aragonite lattice by randomly substituting for Ca [36]. This substitution process was controlled by crystal chemistry and was unlikely to be disturbed by post-mortem degradation or organic decomposition, conferring greater stability than that of other elements. This mechanism provided theoretical support for using desiccated specimens for Sr/Ca ratio analysis. In this study, the specimens included both freshwater resident short-jaw anchovies (22LSGCB01, 22DCGCB01) and anadromous long-jaw anchovies (22LSCN01, 22DCGCN01–08), some of which were incomplete or decayed dried carcasses. Nevertheless, their otolith microchemical signatures clearly distinguished the two ecotypes: short-jaw anchovies showed consistently low Sr/Ca ratios (<3), consistent with a freshwater resident history; long-jaw anchovies exhibited a typical three-phase pattern (SI < 3, SII > 3 and SIII < 3), corresponding to a life history of “freshwater hatching–marine feeding–spawning migration into freshwater”. These characteristics were highly consistent with the Sr/Ca ratios and mapping results of the 2024 fresh individuals (Figure 3 and Figure 4). Although the otolith Sr/Ca records from the drought year were clearly interpretable, the small sample size (*n* = 11 total; *n* = 9 long-jaw migrants) remained a limitation. These specimens represented a rare, opportunistic collection from the exposed lakebed, but the unbalanced sample sizes reduced statistical power and limited the generalizability of the drought-year conclusions. Accordingly, the present results should be viewed as a case study of an extreme event rather than as a comprehensive estimate of population-level responses. The Mann-Whitney *U*-test was used because it does not assume normality or equal sample sizes, but its power is lower with only 11 drought-year individuals. Replication across multiple drought events or with larger sample sizes would be needed to confirm the patterns observed here. In addition, SEM examination revealed no apparent dissolution or recrystallization on the otolith sections of the 2022 desiccated specimens relative to the results for the 2024 fresh specimens (Figure 5). In summary, our results indicated that otoliths from desiccated specimens could preserve life history information with reasonable accuracy, suggesting that this method may be reliable for special sample types. Furthermore, our results indicated that the Sr mapping (Figure 4) corresponded well with the Sr/Ca ratios. Future studies could combine these two features—Sr mapping and annular growth patterns—to further improve ecological research on migratory fish based on otolith microchemistry.

Having confirmed the validity of records from desiccated specimens, we further compared the phase differences between the 2022 and 2024 migratory individuals. The results showed no significant difference in the SII phase (seawater habitat) between the two years (*p* = 0.596), which was consistent with the relatively constant elemental composition of seawater [37], and also indirectly confirmed that the impact of desiccation and decay on Sr/Ca ratios was minimal. However, both the SI and SIII phases showed highly significant differences between the two years (*p* < 0.01), with the SIII phase ratio in 2024 being much higher than in 2022 (Figure 6). Since both SI and SIII correspond to freshwater or low-salinity habitats, where Sr/Ca ratios were easily regulated by climate factors such as terrestrial runoff, precipitation, and evaporation, this difference suggested that the extreme climate might have significantly altered the elemental composition of the freshwater end-member, thereby affecting the chemical record in the corresponding otolith phases.

### 4.2. Differences in Migratory Characteristics of C. nasus Under Extreme and Normal Climate Conditions

Based on the conclusion that desiccated specimens accurately record habitat history, we further used these specimens to compare migratory strategy differences between contrasting climatic years. In this study, except for the two short-jaw anchovies from 2022, all long-jaw anchovy individuals exhibited a typical anadromous migratory pattern of freshwater (SI)–brackish/seawater (SII)–freshwater (SIII) (Figure 3 and Figure 6). As a structure that deposited continuously throughout life and was not subject to bone resorption [16], otolith radius length has been confirmed to be positively correlated with age and is one of the most reliable morphological indicators for age prediction [38,39]. Accordingly, the freshwater coefficient (*Fc*) could effectively reflect the degree of dependence on freshwater habitats during early life history [14]. Comparison of the *Fc* between the two years showed no significant difference (*p* = 0.106, Figure 7). Considering the extremely strong natal homing behavior of *C. nasus* [13,14], the consistency in *Fc* between the two years suggests that both groups in this study may have hatched from the Poyang Lake population. Based on otolith ^87^Sr/^86^Sr signatures, it should be noted that even within the Poyang Lake population, the timing of lake departure varies among individuals [13]. In addition to our results, it is noteworthy that although the 2024 group included 3-year-old individuals (i.e., hatched in 2022), their *Fc* did not differ significantly from that of the 2022 group. This might be because, under extreme drought conditions, larvae entered the Yangtze mainstem earlier, thereby shortening the retention period in the lake and avoiding adverse impacts of the extreme climate on the early freshwater-dependent stage. Future studies could introduce ^87^Sr/^86^Sr isotope tracing to verify the specific timing of lake departure for individuals from different hatching years.

**Figure 8:**
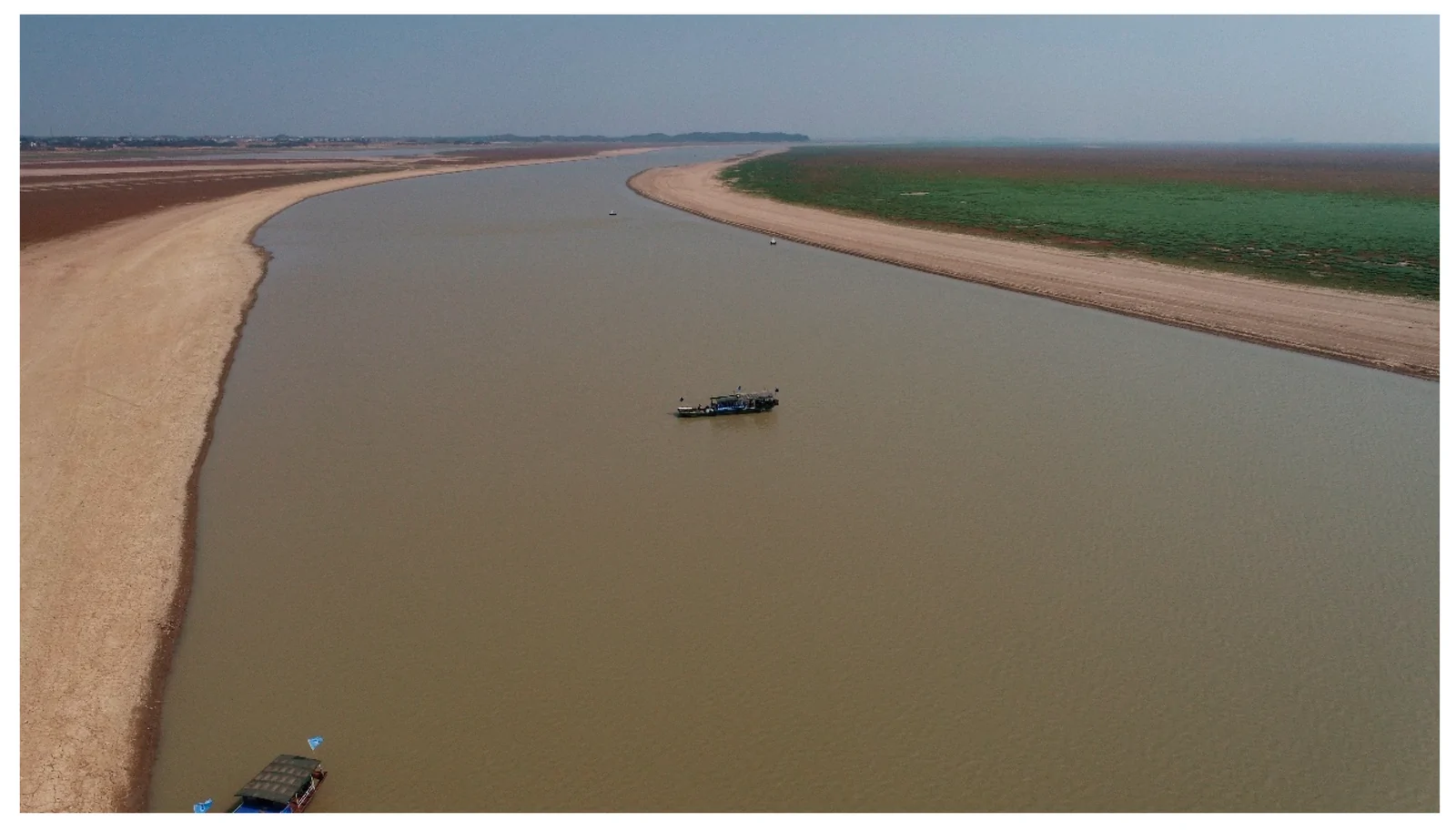
The Duchang waters of Poyang Lake under the influence of the 2022 extreme climate conditions, when the formerly expansive lake area turned into a winding channel due to falling water levels.

In contrast to the non-significant change in the freshwater coefficient, the otolith edge SIII phase length (after entering freshwater) showed a significant difference between the two years. In 2024, this phase was relatively short (33 ± 24 μm, Figure 7), with some individuals even lacking an edge, with the Sr/Ca ratio falling below 3 (e.g., 24DCCN02), implying that they quickly swam upstream into the Poyang Lake waters upon entering freshwater. Similarly, anadromous Atlantic salmon (*Salmo salar* L.) essentially cease feeding and accelerate their upstream migration [40]; *C. nasus* spawners exhibit analogous behavior, with food intake significantly reduced during upstream migration to the spawning grounds [41]. Therefore, although Poyang Lake is over 800 km from the Yangtze Estuary, spawners in normal years relied on homing instinct to migrate rapidly upstream. However, the response of otolith Sr/Ca ratios to salinity changes may not be instantaneous but may involve a certain lag—for example, up to 30–60 days in Japanese eel (*Anguilla japonica*) [42], and weeks to months in brown trout (*Salmo trutta*) [43], with a more pronounced lag when transitioning from high to low salinity [44]. Thus, in normal years, the SIII phase length should be much shorter than the SI phase, and some individuals might still display brackish-water signatures at the edge upon reaching Poyang Lake (Figure 3b). In contrast, the 2022 long-jaw anchovy group, while still showing an SIII phase shorter than the SI phase, exhibited a significantly longer SIII (144 ± 30 μm, Figure 3a and Figure 7). Coupled with the fact that the extreme drought in Poyang Lake that year caused a dramatic drop in water level and shrinkage of the lake area into a channel-like watercourse (Figure 8), the reduced water level likely significantly slowed the upstream migration speed of spawners, forcing them to linger longer in the low-salinity or brackish transition zone, thereby leaving a longer SIII record in the otolith. It should be noted that the longer SIII phase itself indicated prolonged upstream migration and the difficulties faced by adults. Meanwhile, dead spawners were found on the desiccated historical spawning grounds, even though alternative habitats with suitable water were available downstream. All of these findings indicated strong natal homing behavior, meaning that the fish did not divert to alternative spawning sites. Moreover, the mass mortality at the original site revealed the potential vulnerability of such strong homing behavior under extreme climatic events.

Integrating the above analyses, the impact of extreme climate on different life history stages of *C. nasus* was two-fold: for larvae, it might prompt earlier departure from Poyang Lake without significantly altering the overall freshwater dependence; for adults, it severely impeded upstream migration speed due to strong homing behavior, even leading to mass mortality. These findings provided a key ecological basis for formulating subsequent conservation strategies.

## 5. Conclusions

This study confirmed that otolith microchemistry is not only applicable to fresh samples, but it can also clearly reconstruct the habitat history of special specimens such as desiccated and decayed individuals, providing a reliable tool for migratory ecology studies using historical collections and archaeological samples. The majority of the desiccated long-jaw anchovies examined in this study pertained to the 2022 extreme drought event, reflecting both the stock recovery after the fishing ban and the severe consequences of homing behavior when spawning ground function was lost—spawners were unable to find alternative spawning grounds quickly, resulting in population loss. The soon-to-be-constructed Poyang Lake Water Conservancy Hub is expected to mitigate the impact of extreme climate on lake water levels. However, given the strong dependency and low adaptability of *C. nasus* to their historical spawning grounds, the hub operation must clearly define ecological baselines. It is recommended that during the impoundment period, the minimum ecological water level of historical spawning grounds be maintained to strictly prevent desiccation; during the migration windows for upstream spawning and downstream dispersal of larvae, river–lake connectivity must be ensured to avoid blockage by dams. Under the dual pressures of global warming–drying and engineering regulation, incorporating spawning ground protection and migration corridor connectivity into the adaptive operation of the hub is a key measure for maintaining the sustainability of the *C. nasus* population, and it also provides an important reference for the conservation of other aquatic organisms in the lake area.

## Figures and Tables

**Table 1:** Sampling information of dried *Coilia nasus* specimens.

Specimen ID	Sampling Time	Sampling Site ^a^	Total Length (mm)	Body Weight ^b^ (g)	Length of Maxilla (mm)	Length of Head (mm)	Maxilla-to-Head Ratio	Age
22DCGCN01	2022.09	DCW	337	-	53.54	45.49	1.18	3
22DCGCN02	2022.09	DCW	343	-	53.88	46.17	1.17	3
22DCGCN03	2022.09	DCW	296	-	57.5	41.93	1.37	2
22DCGCN04	2022.09	DCW	317	-	54.04	46.97	1.15	3
22DCGCN05	2022.09	DCW	312	-	50.67	42.63	1.19	3
22DCGCN06	2022.09	DCW	296	-	49.4	43.28	1.14	3
22DCGCN07	2022.09	DCW	317	-	50.69	43.87	1.16	3
22DCGCN08	2022.09	DCW	306	-	49.79	41.88	1.19	3
22DCGCB01	2022.09	DCW	292	-	37.5	41.93	0.89	3
22LSGCN01	2022.09	LSW	274	-	45.86	41.83	1.10	2
22LSGCB01	2022.09	LSW	182	-	26.52	29.98	0.88	2
24DCCN01	2024.08	DCW	317	105.3	54.04	46.97	1.15	3
24DCCN02	2024.08	DCW	343	106.56	52.84	45.94	1.15	3
24DCCN03	2024.08	DCW	317	77.04	50.69	43.87	1.16	3
24DCCN04	2024.08	DCW	337	100.48	53.29	48.53	1.10	3
24DCCN05	2024.08	DCW	329	88.62	49.12	42.79	1.15	3
24DCCN06	2024.08	DCW	321	85.06	48.02	41.54	1.16	3
24DCCN07	2024.08	DCW	326	96.03	54.28	45.97	1.18	3
24DCCN08	2024.08	DCW	367	111.32	60.87	50.96	1.19	4
24DCCN09	2024.08	DCW	337	95.02	53.28	46.17	1.15	3
24DCCN10	2024.08	DCW	357	92.65	56.5	48.18	1.17	4
24DCCN11	2024.08	DCW	337	82.03	51.38	46.44	1.11	3
24DCCN12	2024.08	DCW	367	112.87	57.11	47.36	1.21	4
24DCCN13	2024.08	DCW	361	158.04	54.44	50.41	1.08	4
24DCCN14	2024.08	DCW	312	70.62	51.59	42.06	1.23	3
24DCCN15	2024.08	DCW	324	87.35	51.17	44.49	1.15	3
24DCCN16	2024.08	DCW	322	80.66	51.52	44.4	1.16	3
24DCCN17	2024.08	DCW	312	80.59	50.67	42.63	1.19	3
24DCCN18	2024.08	DCW	306	53	49.79	41.88	1.19	3

^a^: DCW: Duchang County waters; LSW: Lushan City waters; ^b^: Body weight of dried specimens collected in 2022 was not measured due to incomplete body condition.

## Data Availability

Data is contained within the article.
